# A versatile method for the preparation of particle-loaded microbubbles for multimodality imaging and targeted drug delivery

**DOI:** 10.1007/s13346-017-0366-7

**Published:** 2017-03-15

**Authors:** Joshua Owen, Calum Crake, Jeong Yu Lee, Dario Carugo, Estelle Beguin, Alexandre A Khrapitchev, Richard J Browning, Nicola Sibson, Eleanor Stride

**Affiliations:** 1 Institute of Biomedical Engineering, Department of Engineering Science, Old Road Campus Research Building, University of Oxford, Headington, Oxford, OX3 7DQ UK; 2 Present Address: Department of Radiology, Brigham and Women’s Hospital, Harvard Medical School, 221 Longwood Avenue, Boston, MA 02115 USA; 3 Present Address: Faculty of Engineering and the Environment, University of Southampton, Southampton, UK; 4 Cancer Research UK & Medical Research Council Oxford Institute for Radiation Oncology, Department of Oncology, Old Road Campus Research Building, University of Oxford, Headington, Oxford, OX3 7DQ UK

**Keywords:** Microbubbles, Ultrasound, Contrast agents, Magnetic, Targeting, Drug delivery

## Abstract

Microbubbles are currently in clinical use as ultrasound contrast agents and under active investigation as mediators of ultrasound therapy. To improve the theranostic potential of microbubbles, nanoparticles can be attached to the bubble shell for imaging, targeting and/or enhancement of acoustic response. Existing methods for fabricating particle-loaded bubbles, however, require the use of polymers, oil layers or chemical reactions for particle incorporation; embed/attach the particles that can reduce echogenicity; impair biocompatibility; and/or involve multiple processing steps. Here, we describe a simple method to embed nanoparticles in a phospholipid-coated microbubble formulation that overcomes these limitations. Magnetic nanoparticles are used to demonstrate the method with a range of different microbubble formulations. The size distribution and yield of microbubbles are shown to be unaffected by the addition of the particles. We further show that the microbubbles can be retained against flow using a permanent magnet, can be visualised by both ultrasound and magnetic resonance imaging (MRI) and can be used to transfect SH-SY5Y cells with fluorescent small interfering RNA under the application of a magnetic field and ultrasound field.

**Electronic supplementary material:**

The online version of this article (doi:10.1007/s13346-017-0366-7) contains supplementary material, which is available to authorized users.

## Introduction

The first use of microbubbles as contrast agents for ultrasound imaging dates back to the 1960s. However, early formulations were unstable and difficult to reproduce. It was found that a coating of albumin, initially derived from a patient’s own blood, improved stability in vivo [1]. Since then, other types of coating have been investigated, leading to a number of commercial products including albumin-coated microbubbles such as Albunex^®^ and Optison^®^ (GE Healthcare), microbubbles formed from the dissolution of galactose crystals and stabilised by palmitic acid such as Echovist^®^ and Levovist^®^ (Schering AG) [2] and phospholipid-coated microbubbles such as SonoVue^®^ (Bracco), Sonazoid^®^ (GE Healthcare) and Definity^®^ (Lantheus Medical Imaging) [3]. New agents are also being developed based on gas nanostructures formed by certain bacteria and archaea [4]. A number of polymers have also been investigated for stabilising microbubbles, for example polysaccharide alginate by Wheatley et al. [5], arginine by Florinas et al. [6] and poly(allylamine hydrochloride) grafted onto albumin by Lentacker et al. [7]. Polymerisation of material around the gas core produces a stiff bubble that has excellent stability but comparatively poor acoustic response; to date, there have been no clinically approved polymer-based agents [5, 8].

Microbubbles are also being widely investigated for therapeutic applications. They can be used as nuclei to increase cavitation activity [9] and/or as vehicles for drug delivery/gene therapy by incorporation of therapeutic material which can be subsequently released by destroying the microbubbles with a high-amplitude ultrasound pulse [10–12]. The activity of microbubbles produced by ultrasound exposure can improve the penetration of a drug into a tissue volume [13–15] and reversibly permeabilise cell membranes [16–19]. For example, microbubbles have been shown to increase the uptake of drugs such as paclitaxel and doxorubicin as well as colloidal particles [11, 20, 21] and this is still a very active area of research [22, 23].

Their ability to both improve ultrasound contrast and deliver therapeutic molecules makes microbubbles excellent candidates as theranostic agents. Their functionality can be further enhanced through the incorporation or attachment of nanoparticles to the microbubble shell. For example, superparamagnetic iron oxide nanoparticles (SPIONs) that are used clinically as MRI contrast agents [24, 25] can be used to enable both multimodality imaging and magnetic targeting of microbubbles. Gold nanoparticles can be attached to impart photoacoustic contrast enhancement [26, 27]. Drugs can also be loaded onto the microbubble shell in the form of particles [21].

Incorporating nanoparticles into polymer microbubbles usually involves adding the nanoparticles during the formation of the microbubble so that they become embedded in the polymer shell. For example, lysozyme and PVA microbubbles have been created with gold nanoparticles embedded in their surface via a pressurised gyration process for drug delivery and biosensing [27]. Yang et al. prepared a microbubble construct loaded with SPIONs for dual contrast imaging [28]. Park et al. have similarly produced stable microbubbles which can attach different types of nanoparticles to their surface for use in image contrast enhancement [29]. He et al. developed a polymer magnetic microbubble for MRI with iron oxide nanoparticles on the surface of the microbubble which improved the non-linear acoustic characteristics [30]. Duan et al. used a synergistic approach of covalent coupling, electrostatic adsorption and aggregation to attach nanoparticles to polymer microbubbles for determination of optimum loading [31]. Self-assembled polymer microcapsules have also been developed via sonication of poly(allylamine) and poly(acrylic acid) solution with citric acid-coated iron oxide nanoparticles for dual-MRI and ultrasound contrast enhancement [32].

Kovalenko et al. developed hollow magnetic microspheres using surfactant microbubbles and adsorbed cobalt ferrite nanoparticles [33]. Silver nanoparticles have also been embedded into polymer microbubble shells for dark field microscopy again by coating them with polymer and incorporating them into the microbubble shell during the production process [34]. *Upconversion* nanoparticles have also been attached to the surface of microbubbles through a layer-by-layer approach for fluorescence and ultrasound imaging [35].

Nanoparticles can be attached to phospholipid-coated microbubbles via a variety of methods including biochemical conjugation, electrostatic interaction or the addition of a hydrophobic carrier layer between the gas core and surfactant coating. Mohamedi et al. [36] used a simple physical adsorption method in order to attach gold nanoparticles to the surface of phospholipid-coated microbubbles. This was found to improve the stability and non-linear acoustic response of the microbubbles [36, 37]. Gold particles have also been attached to microbubbles using chemically modified phospholipids, e.g. by using a thiol linkage [38] or using avidin-coated gold nanoparticles and biotinylated microbubbles, in both cases for photoacoustic imaging [26, 39].

Phospholipid-coated magnetic microbubbles consisting of a gas core surrounded by a layer of ferrofluid (liquid hydrocarbon suspension of iron oxide nanoparticles) were successfully used for gene delivery by Stride et al. [40] and Mulvana et al. [41]. The ferrofluid did, however, reduce the acoustic responsiveness of the microbubbles, and there were some concerns regarding the biocompatibility of the carrier liquid. In 2010, Mykhaylyk et al. [42] and Vlaskou et al. [43] published a study on magnetically and acoustically active lipospheres. Lipospheres differ from microbubbles as they contain a bilayer stabilised by a polar fluid rather than a monolayer at the gas–water interface. The lipospheres were synthesised by mechanical agitation of a mixture of soybean oil, a cationic lipid, magnetic nanoparticles, DNA, aqueous buffer and a perfluoropropane gas in a sealed vial [43]. As therapeutic delivery agents, the lipospheres did enable nucleic acid delivery under a magnetic field but the soybean oil again reduced the acoustic responsiveness of the particles and there was no increase in cell membrane permeability when ultrasound and a magnetic field were applied [43]. Vlaskou et al. [43, 44] subsequently investigated lipid shell microbubbles conjugated with positively charged magnetic nanoparticles through electrostatic interactions. These microbubbles had a much greater response to ultrasound, and a combination of ultrasound and magnetic forces improved transfection efficiency in vitro and in vivo [44, 45]. Uncoated iron oxide nanoparticles have also been attached to lipid microbubbles by vigorous shaking for targeting magnetic stents, although the mechanism of incorporation in this study was not investigated in detail [46].

Magnetic nanoparticles have also been used in order to augment biomarker targeting. In 2011, magnetic microbubbles were targeted via vascular cell adhesion molecule 1 (VCAM-1) for molecular imaging and a magnetically targeted microbubble system resulted in greater attachment to VCAM-1 in atherosclerotic aortas in mice in conditions of high fluid shear stress. VCAM-1 was attached via avidin–biotin, and iron oxide nanoparticles were also attached to the avidin bound to the bubble [47].

Unfortunately, both electrostatic and biomimetic reactions, such as avidin–biotin conjugation techniques, and hydrophobic layers also pose challenges in terms of their ultimate clinical use. Charged particles have been shown to trigger unwanted immune responses [48], while avidin–biotin conjugation is time consuming with poor biocompatibility [49] and hydrophobic layers can dampen the acoustic response of microbubbles [41, 43]. The aim of this study was to develop a method of incorporating nanoparticles into phospholipid shells in order to allow for adaptation of clinically approved microbubble formulations.

## Materials and methods

### Materials

1,2-Distearoyl-*sn*-glycero-3-phosphocholine (DSPC), 1,2-dipalmitoyl-*sn*-glycero-3-phosphocholine (DPPC), 1,2-dibehenoyl-*sn*-glycero-3-phosphocholine (DBPC), 1,2-distearoyl-*sn*-glycero-3-ethylphosphocholine (DSEPC), 1,2-distearoyl-*sn*-glycero-3-phosphoethanolamine-*N*-(polyethylene glycol)-2000 (DSPE-PEG(2000)) and 1,2-distearoyl-*sn*-glycero-3-phosphoethanolamine-*N*-[biotinyl(polyethylene glycol)-2000] (DSPE-PEG(2000)-biotin) were purchased from Avanti Polar Lipids, Inc. (Alabaster, AL, USA). Polyethylene glycol (PEG)-40 stearate, ethanol, chloroform, Dulbecco’s phosphate-buffered saline, foetal bovine serum, glycerol, propylene glycol, avidin, fluorescein isothiocyanate (FITC) avidin, biotin and agarose powder were purchased from Sigma-Aldrich Ltd. (Gillingham, Dorset, UK). Phospholipid (phosphatidylcholine)-coated 50-nm magnetite nanoparticles (FluidMAG-Lipid) were purchased from Chemicell GmbH (Berlin, Germany). Sulphur hexafluoride (SF_6_) was purchased from The BOC Group (Guilford, Surrey, UK).

BLOCK-iT™ Fluorescent Oligo (Lot No. 1477937) was purchased from Invitrogen (Life Technologies). This is a generic small interfering RNA (siRNA) with a fluorophore attached that does not cause knockdown of a specific gene. Phenol-free Dulbecco’s modified Eagle’s medium (DMEM) and trypsin–EDTA (0.05%) phenol red were also purchased from Life Technologies, Inchinnan Business Park, Paisley, UK.

SH-SY5Y neuroblastoma cells (from ATCC from LGC Standards, UK Office, Teddington, UK) were used as a model cell line because of their rapid replication rate and ability to be transfected using viral methods. Neuroblastoma is also one the most common extracranial solid tumours in children with certain tumours expressing resistance to multimodal treatment [50]; thus, the development of potential treatment methods is highly desirable.

### Bubble manufacturing technique

For the initial experiments, five different microbubble formulations were utilised to test the versatility of the technique. For each formulation, the lipids were dissolved in chloroform, mixed in a glass vial at a selected molar ratio (Table 1), heated to 50 °C and left for 12 h to evaporate the solvent. The resulting lipid film was then suspended in aqueous solution (2 ml at a concentration of 15 mg/ml) for ~1 h at 75 °C under constant stirring as above. The stir bar was removed, and the solution was then sonicated using an ultrasonic cell disruptor (XL2000, probe diameter 3 mm; Misonix, Inc., Farmingdale, NY, USA) operating at 22.5 kHz and level 4 corresponding to 8 W_RMS_ output power, for 90 s, followed by sonication at the gas–water interface (ensuring the probe tip touches the water surface) under positive pressure of SF_6_ for 20 s at sonication power 19 (38 W_RMS_). The microbubble solution was then placed in an ice bath for cooling for approximately 10 min.

**Table 1:** Different microbubble formulations and the corresponding molar ratio of components used in the present study

Name	Components	Molar ratio
(i) Short chain	DPPC, PEG-40 stearate	9:1
(ii) Medium chain	DSPC, PEG-40 stearate	9:1
(iii) Long chain	DBPC, PEG-40 stearate	9:1
(iv) Charged	DSPC, DSEPC, PEG-40 stearate	100:44:4.5
(v) Targeting	DSPC, DSPE-PEG(2000), DSPE-PEG(2000)-biotin	82:9:9

To prepare the magnetic microbubbles from each of the formulations, after 60 s of sonication (power setting 4), a suspension of (FluidMAG-Lipid) phosphatidylcholine-coated magnetic nanoparticles in water (150 μl, 25 mg/ml) was added. Sonication continued for a further 30 s followed by sonication at the gas–water interface under a positive pressure of SF_6_ for 20 s at power setting 19 as mentioned above.

### Microbubble characterisation

#### Size distribution and concentration

Two methods were used for determining the size distribution and concentration of the microbubble suspensions. The first was single particle optical sizing (SPOS) using an AccuSizer 780 (NICOMP Particle Sizing Systems, Santa Barbara, CA, USA) in which a 10 μl sample of each microbubble suspension was diluted in 50 ml of filtered deionised water in a flask under mild mixing during measurement. The second was direct observation under an optical microscope [51]. In the latter method, 10 μl of the microbubble suspension was injected into a haemocytometer. This was then observed using a Leica DM500 optical microscope (Larch House, Milton Keynes, MK14 6FG) with a ×40 objective lens at room temperature. The bubble size distribution and concentration were obtained using purpose written image analysis software in MATLAB (2013b, The MathWorks, Natick, MA, USA). The software converts each micrograph into a binary image, and single circular shapes are then located, measured and counted for each of the images analysed. A size distribution and count are then produced for the microbubbles. In order to obtain a representative size distribution for a single batch of bubbles, at least 30 images from three separate bubble samples must be analysed [51].

#### Stability over time

Microbubble stability was determined by recording their size distribution and concentration over 3 h using the second method described above of direct observation under an optical microscope and MATLAB image analysis. To enable a comparison between formulations and different samples, both the microbubble mean diameter and concentration were non-dimensionalised with respect to the initial value at the start of the experiment for each sample. Bubbles made from the medium chain formulation with and without magnetic nanoparticles were also characterised at both the ambient and physiologically normal temperatures of 21 and 37 °C, respectively.

#### Optical observation of magnetic response

A simple initial test was used to determine whether microbubbles had been successfully functionalised with magnetic nanoparticles. An N45 NdFeB permanent magnet (40 mm × 18 mm × 10 mm) with a magnetic field strength of 0.365 T at a distance of 0.7 mm from the surface of the magnetic pole was held at the surface of the vial containing the microbubbles in order to observe whether or not microbubbles would respond.

#### Transmission electron microscopy

The medium chain formulation was subjected to further, more detailed characterisation. Transmission electron microscopy (TEM) was used to investigate the surface structure of individual microbubbles and to confirm the incorporation of nanoparticles [52]. Five microlitres of each microbubble suspension was applied to a freshly glow-discharged carbon-coated copper grid, and the grid was inverted for approximately 1 min in order to allow a high concentration of microbubbles to remain in contact with the grid. The sample was then negatively stained with 2% *w*/*v* uranyl acetate. Samples were visualised at 80 kV with an FEI Tecnai™ T12 electron microscope. Low-dose images were acquired at ~0.8 μm underfocus with 15*e* ^−^/Å on a high-sensitivity FEI Eagle 4096 × 4096 pixel CCD camera at a nominal magnification of ×46,000 which corresponded to a sampling size of 0.265 nm/pixel. On average, 10 microbubbles were analysed for each sample. Microbubbles were excluded if they appeared agglomerated such that one microbubble could not be differentiated from another. No other objects of the same size were observed in the suspensions.

#### Magnetic relaxivity

The magnetic relaxivity was also measured for the same microbubble formulation as this provides an indication of responsiveness to a magnetic field and potential utility as an MRI contrast agent. Measurements were performed using a 4.7 T Magnex or 7.0 T superconductive magnet driven by a Varian DirectDrive™ spectrometer (Magnex Scientific and Varian, Inc., subsidiaries of Agilent Technologies, Santa Clara, CA, USA). A spin echo sequence was used to acquire T_2_ and T_1_ maps. Single slice images were acquired with a matrix size of 128 × 128 pixels in all cases, corresponding to voxel dimensions of 0.4 × 0.4 × 5.0 mm. T_2_ maps were generated from a series of spin echo images (repetition time (TR) = 3.0 s) in which the echo time (TE) was logarithmically distributed in 10 steps from 9.7 to 100 ms. The total experimental time was ~1 h. T_1_ maps were generated from a series of inversion recovery spin echo images (TR = 10.0 s; TE = 9.7 ms) in which the inversion recovery time was logarithmically distributed in 10 steps from 10 ms to 6.0 s. The total experimental time was ~3.5 h. The relaxation maps were calculated using a standard mono-exponential fit employing a least squares procedure.

#### Magnetic targeting under flow

To investigate magnetic targeting under flow, ultrasound imaging was used to observe the microbubbles. Details of the apparatus are provided in the study of Owen et al. [53], but briefly, a latex tube (3 mm inner diameter) was suspended in a water bath at ambient temperature (23 °C) and connected to a peristaltic pump (Gilson MiniPuls 3, Gilson, Luton, Bedfordshire, UK) drawing from a reservoir of the relevant suspending liquid. A section of the tube was positioned so that it was parallel to the base of the bath with a gap of approximately 3 cm to allow for the insertion of a magnetic Halbach array consisting of five rectangular block N52 magnets (10 mm × 10 mm × 25 mm, supplied by NeoTeXx, Berlin, Germany) with transversal magnetisations (1.5 T) oriented at angles of 90° from one to the next in an aluminium frame. A T junction was connected to the tubing to allow for the injection of magnetic microbubbles upstream of the magnet. The outlet of the tubing was fed to a waste reservoir at atmospheric pressure (supplementary Fig. 1).

An ultrasound linear array transducer (9.4 MHz LA523, Esaote, Italy) was positioned above the section of tube under which the magnetic array was located in order to visualise the microbubbles. Video sequences were acquired using an ULA-OP ultrasound engine (Microelectronic System Design Lab., Universita degli Studi di Firenze, Firenze, Italy). The flow rate was selected based on a previous study of magnetic targeting of microbubbles against in vivo flow conditions [53]. Once a steady flow had been established (0.25 ml/s) in the tube, data were acquired for a few seconds to provide a baseline image and a 1.5 ml bolus of magnetic microbubbles was then injected and data were acquired for a further 60 s. This process was repeated three times with and without a magnetic field applied.

### Delivery of therapeutic compounds

Further tests were also performed to investigate the therapeutic potential of magnetic microbubbles using the charged microbubble formulation. For these tests, the charged bubble formulation was used to facilitate attachment of siRNA.^1^ This was based upon the work of Carson et al. [54], and the aim was to show the delivery capabilities of the microbubbles under ultrasound and a magnetic field using an existing platform.

#### Zeta potential

Measurements of lipid vesicles and microbubbles (separately) were performed via dynamic light scattering using a Zetasizer Nano ZS, Malvern Instruments Ltd. (Worcestershire, UK) in a disposable capillary cell (DTS1070) from Malvern Instruments Ltd. (Worcestershire, UK). Each sample of the vesicle or microbubble solution (60 μl) was added to 940 μl of 10% HEPES buffer and measured using the Smoluchowski protocol for up to 100 runs in order to produce an accurate zeta potential measurement.

#### Attachment of siRNA

Twenty microlitres (7 μg) of fluorescently labelled siRNA was added to the lipid solution before sonication at the gas–water interface. The solution was sonicated for 10 s followed by sonication at the air–water interface under SF_6_ as described previously. The solution was then washed by centrifugation (300 relative centrifugal force (RCF), 10 min) in order to remove any excess siRNA [54]. This was then analysed via fluorescence microscopy in order to determine whether the siRNA had successfully attached to the microbubble surface. The amount of siRNA that attached is based on the work of Carson et al. [54] where it was determined that the maximum amount of siRNA which can be attached to 1 × 10^9^ microbubbles (approximately the number of microbubbles per ml) is 7 μg. This was the quantity of siRNA added to the bubble mixture and was equivalent to 7 × 10^−9^ μg of siRNA per microbubble. The charge was used to attach the siRNA and was not desirable for use in vivo; however, it is currently the optimal method for attachment of siRNA to the microbubble.

#### Cell culture

SH-SY5Y cells were examined for the delivery of fluorescent siRNA. The cells were cultured in flasks using phenol-free DMEM with FCS and antibiotics. When the cells were at confluence, they were trypsinised, centrifuged and distributed between OptiCell™ chambers (Thermo Fisher Scientific, Bishop Meadow Road, Loughborough, UK) in phenol-free DMEM. This resulted in a final concentration of 1.5 × 10^7^ cells per OptiCell™. These were allowed to reach confluence overnight in an incubator at 37 °C.

#### Ultrasound exposure

**Fig. 1:**
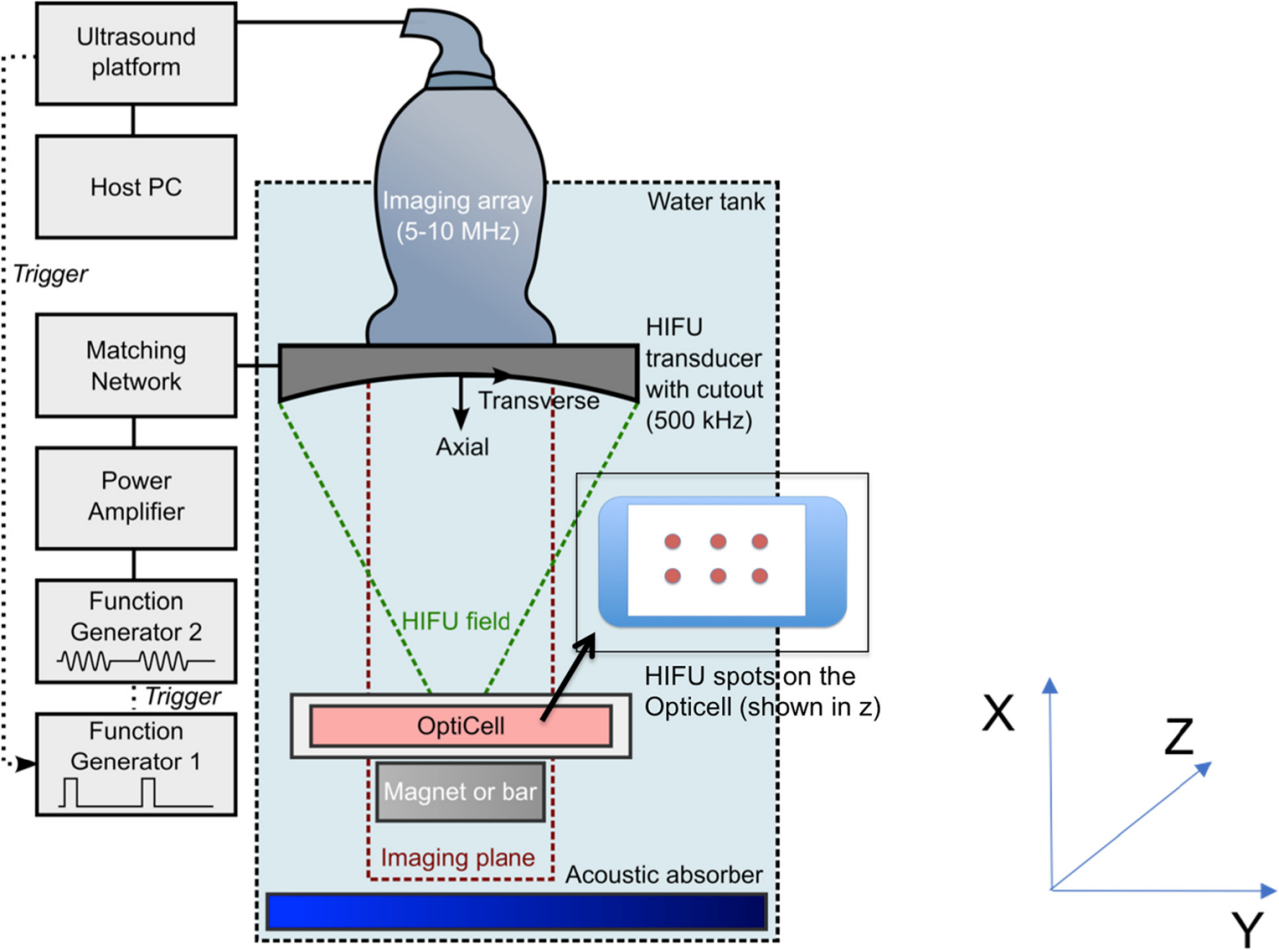
Setup for cell transfection experiments (not to scale). A cross-sectional view through the water tank is shown. Samples were placed in a cell chamber at the focus of a 500-kHz focused ultrasound transducer with a rectangular cutout for an imaging array. The imaging array was used for B-mode imaging for alignment of the sample at the focus and to passively record acoustic emissions during ultrasound exposure. The transducer was driven by a function generator via a power amplifier and impedance matching network. A second function generator set to generate a pulse train was used to allow treatment to take place at a multiple of the imaging frame rate. The *black arrow* points to an OptiCell™ in the *z*-direction, showing the six sites on the cell culture plate that were exposed to the conditions outlined below

A schematic of the setup used for transfection experiments is shown in Fig. 1. Ultrasound was generated using a circular 500-kHz focused ultrasound transducer (model H-107B-10; Sonic Concepts, Inc., Bothell, WA, USA) featuring a rectangular cutout through which a 128-element imaging array (model L10-5; Zonare Medical Systems, Mountain View, CA, USA) was aligned as described previously [55]. The transducer was driven via two function generators (model 33250A; Agilent, Wokingham, Berkshire, UK)—the first was set to pulse mode and triggered from the ultrasound platform—and used to trigger the second which produced the drive signal for the transducer. The use of two function generators thus allowed treatment to occur at a multiple of the imaging frame rate as each imaging frame triggered a chain of treatment pulses. The driving signal was applied to the transducer via a 55-dB power amplifier (model A300; Electronics and Innovation, Rochester, NY, USA) and impedance matching network supplied by the transducer manufacturer. The free-field focal pressure and beam profile of the transducer were calibrated in water using a 400-μm-diameter needle hydrophone (model Onda 1056; Onda Corporation, Sunnyvale, CA, USA). Pressure values referred to subsequently are peak negative focal pressures (PNFPs). The ultrasound platform (model Z-one; Zonare Medical Systems, Mountain View, CA, USA) was connected to a host desktop PC to configure the system for passive acquisition and save data after experiments.

The ultrasound transducer and imaging array assembly was mounted on a three-axis positioning stage in a Perspex tank filled with filtered, degassed, deionised water which was heated to 37 °C using an immersion heater. Prior to commencing experiments, the focus of the transducer was located using a 200-μm needle hydrophone (Precision Acoustics, Dorchester, UK) and the excitation voltage was adjusted to obtain the peak-to-peak focal pressure required for experiments. The location of the hydrophone tip observed under B-mode imaging was recorded and used to position samples at the transducer focus during experiments.

Samples were placed in a cell chamber (OptiCell™; Fisher Scientific, Loughborough, UK) and mounted on top of the Halbach array described above or a non-magnetic aluminium bar of the same dimensions so that any reflections of the ultrasound field would be consistent between experiments. The magnet and chamber assembly was then aligned with the focus of the transducer under B-mode guidance. Based on previous transfection experiments [40], the ultrasound conditions used for treatment were 1 MPa peak-to-peak acoustic pressure, 40 cycles per burst and 1 kHz pulse repetition frequency for 10 s per treatment location. The ultrasound platform was set to passively record the acoustic emissions from cavitation received on the array during experiments at a frame rate of 50 Hz for subsequent mapping and analysis as described below.

Immediately prior to each experiment, 1 ml of a siRNA-loaded microbubble suspension or 1 ml PBS was added to each cell chamber containing a confluent layer of cells on one membrane in DMEM. Microbubbles were dispersed by gentle agitation, and the chamber was placed in the water tank with the cells on the lower surface so that only magnetic microbubbles, upon application of a magnetic field, would be attracted towards them. Each cell chamber was treated at six evenly spaced locations as shown in Fig. 1. A total of five cell chambers were treated using the conditions outlined in Table 2.

**Table 2:** Summary of experimental conditions for transfection experiments

Label	MMB (ml)	Magnet (T)	Ultrasound
OptiCell™ 1	1	–	–
OptiCell™ 2	1	0.5	–
OptiCell™ 3	1	0.5	6 × (1 MPa p-p, 40 cycles, 1 kHz PRF, 10 s)
OptiCell™ 4	1	–	6 × (1 MPa p-p, 40 cycles, 1 kHz PRF, 10 s)
OptiCell™ 5	–	–	6 × (1 MPa p-p, 40 cycles, 1 kHz PRF, 10 s)

*MMB* magnetic microbubbles with siRNA, *p-p* peak-to-peak (focal pressure), *PRF* pulse repetition frequency

Following treatment, the cell chambers were returned to an incubator for approximately 1 h, after which the medium containing microbubbles was removed. The chambers were then washed with media (10 ml) and re-filled with further media (15 ml) followed by analysis via fluorescence microscopy and an automatic plate reader (described below), which was conducted on the same day as treatment.

#### Passive acoustic mapping

Following each experiment, the acoustic emissions captured by the imaging array were mapped in space to provide an estimate of the acoustic power of cavitation emissions using the reconstruction algorithm described in [56]. Maps over a 10 × 20 mm area about the ultrasound focus were generated for each frame of the received data. The sum of these maps from each experiment was used to estimate the total energy of acoustic emissions from each exposure and thus gives a quantitative indication of cavitation activity occurring in each sample.

#### Fluorescence measurements

Fluorescence images were obtained on a Nikon Ti fluorescence microscope using the FITC filter settings. Bubbles placed in an OptiCell™ were analysed in order to determine the conditions under which the fluorescent siRNA could be observed. Cells were examined with the same imaging settings (2 s exposure and gain of 1). Bright-field images were also taken with an exposure time of 363 ms and a gain of 1.

In each area exposed to ultrasound, bright-field (×10) and FITC images were captured at the same location. A large stitched image of 5 mm × 5 mm was obtained at each insonation location in the OptiCell™. Each fluorescence image was analysed in ImageJ in order to obtain an intensity histogram. The maximum intensity in the image was then recorded. For each OptiCell™, an average and a standard deviation of the maximum intensities were determined. The maximum intensity was chosen, owing to the chaotic nature of cavitation-enhanced delivery and inhomogeneous distribution over the insonation location. Differences in the average were likely be small whereas the maximum in the OptiCell™ where transfection has been successful would likely be higher. The average intensity for the whole OptiCell™ was obtained in a BMG Labtech FLUOstar Omega plate reader and processed using the FITC OptiCell™ setting. Each run took 14 min and 22 s. The fluorescence intensity of the whole OptiCell™ was then analysed.

## Results and discussion

**Fig. 2:**
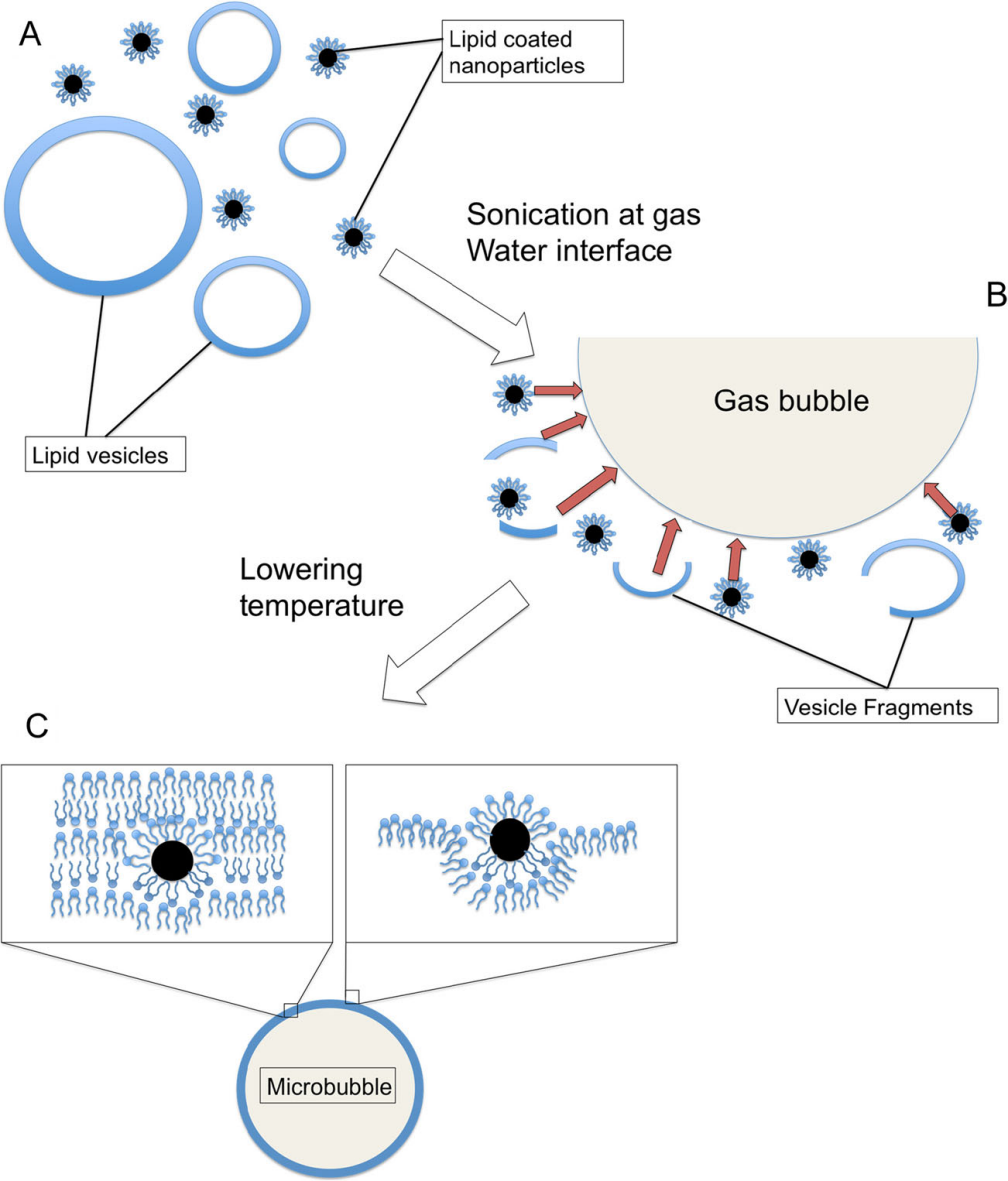
Proposed mechanism of magnetic microbubble formation. *A* A solution with lipid vesicles of varying sizes and lipid-coated nanoparticles is heated above the phase transition temperature. *B* During sonication at the gas–water interface, gas is entrained forming bubbles and the lipid vesicles break up into fragments which adsorb onto the bubbles with the nanoparticles. *C* After cooling, the phospholipid shell condenses with nanoparticles entrapped within it

We have shown in previous work using both fluorescence microscopy and TEM that adsorption onto the microbubble surface of nanoscale vesicles suspended in the liquid surrounding the microbubbles may occur if the vesicle material is chosen such that this process is thermodynamically favourable [52]. This process is illustrated in Fig. 2. It was therefore hypothesised that, through appropriate surface functionalisation, nanoparticles could be incorporated into the microbubble shell through a similar process during microbubble formation. This would avoid the need for additional carrier layers or complex conjugation strategies and should be applicable to a wide range of phospholipid formulations and nanoparticles. For the purposes of this study, superparamagnetic iron oxide was selected as a model nanoparticle since; as mentioned above, this can be used to facilitate both targeting and multimodality imaging.

### Size distribution and concentration

Magnetic microbubbles were successfully produced with all formulations. The results of the sizing and concentration measurements are summarised in Table 3, and the representative images are shown in Fig. 4. The average size (diameter) and concentration did not appear to be adversely affected by the addition of magnetic nanoparticles. The results showed that the addition of magnetic nanoparticles had a minimal effect on the modal average size (within the standard deviation) and similar standard deviations were observed. Changes in concentration were also within the standard deviation for all samples with and without magnetic nanoparticles. Quantitatively different results were obtained from the analysis of the microscope images. However, this was not unexpected based on the results of previous studies [51]. The minimum particle size that can be accurately resolved by the AccuSizer is 0.5 μm, whereas the resolution of a bright-field optical microscope image is limited to ~1 μm. Thus, for populations containing a high proportion of small bubbles (such as the medium and long chain bubbles), the accuracy of the microscope measurements will be reduced.

**Table 3:** Average size and concentration of all bubble formulations with and without magnetic nanoparticles from three separate bubble samples obtained via single particle optical sizing (SPOS) and average size of all bubble formulations via image analysis of optical micrographs

Bubble	AccuSizer				Microscope	
	Modal diameter (μm)	Standard deviation (μm)	Concentration (×10^8^/ml)	Standard deviation (×10^8^/ml)	Modal diameter (μm)	Standard deviation (μm)
Medium chain	0.90	1.31	1.73	0.46	1.65	1.32
Magnetic	1.24	1.92	1.47	0.32	1.56	1.21
Long chain	0.94	1.46	0.38	0.20	2.41	1.65
Magnetic	1.49	2.60	0.59	0.17	2.82	1.90
Short chain	2.92	2.11	0.68	0.23	2.41	1.04
Magnetic	3.86	2.88	0.46	0.14	3.57	1.59
Charged	3.73	3.24	0.41	0.08	2.97	1.87
Magnetic	2.75	2.37	0.70	0.16	2.63	1.52
Targeting	1.07	1.93	1.3	0.42	1.88	1.52
Magnetic	1.02	1.15	1.3	0.30	1.54	0.97

### Bubble stability

**Fig. 3:**
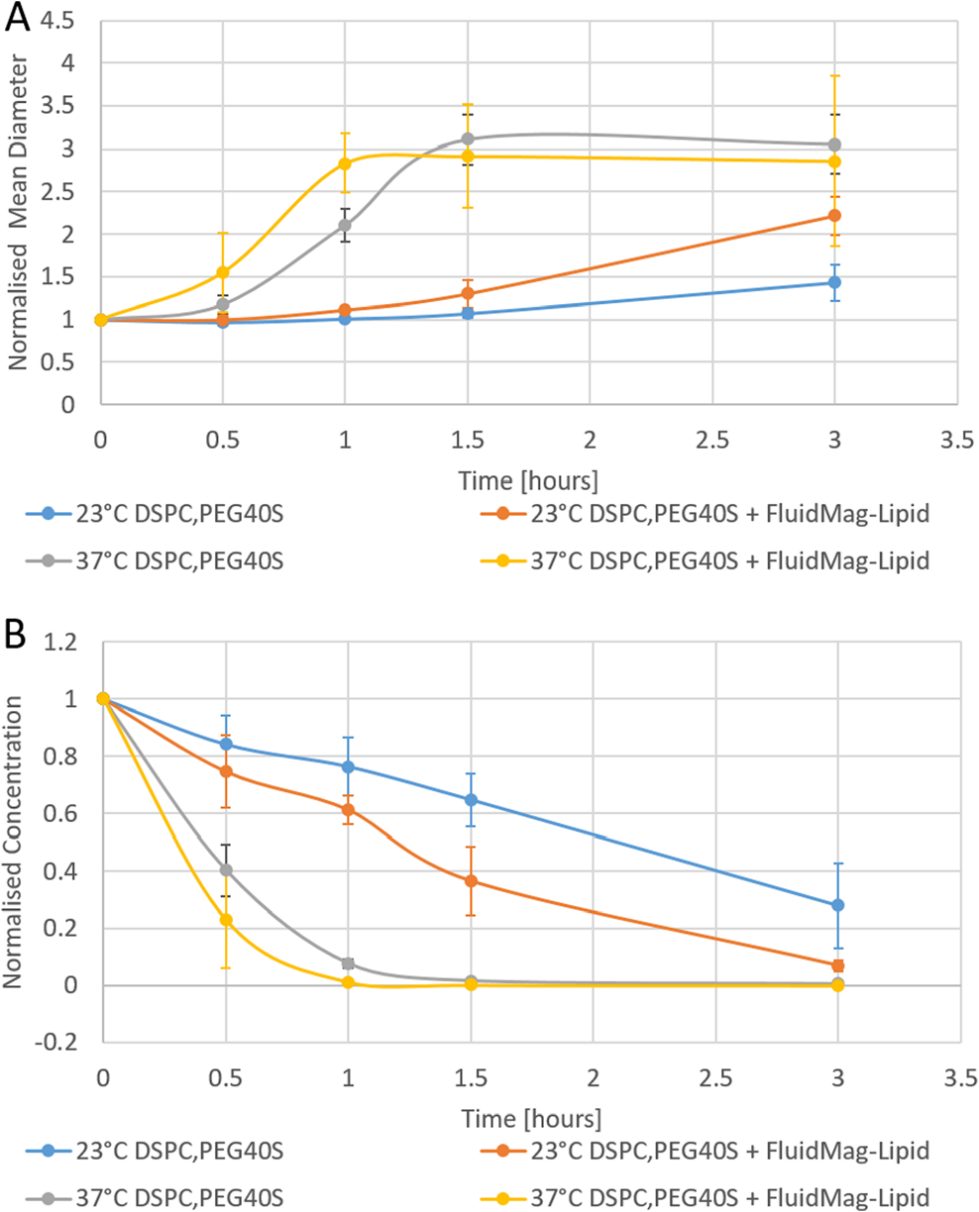
Comparison of microbubble stability with and without iron oxide nanoparticles. Normalised mean diameter and concentration data were calculated for each time point by dividing the measurement result with the initial measurement result at the start of the stability study. **a** Change in mean diameter with time at 23 and 37 °C. **b** Change in concentration with time at 23 and 37 °C. *Error bars* indicate the standard deviation (*n* = 3)

Microbubble stability was assessed by examining changes in size and concentration over time. The results for the medium chain formulation with and without magnetic nanoparticles are presented in Fig. 3. The addition of FluidMAG-Lipid magnetic nanoparticles to microbubbles did not change the stability of the formulation (differences were within the measurement uncertainty). However, an increase in temperature to 37 °C led to a reduction in stability as would be expected. This occurred for non-magnetic and magnetic microbubbles, indicating magnetic nanoparticles do not adversely impact microbubble stability.

### Magnetic targeting

**Fig. 4:**
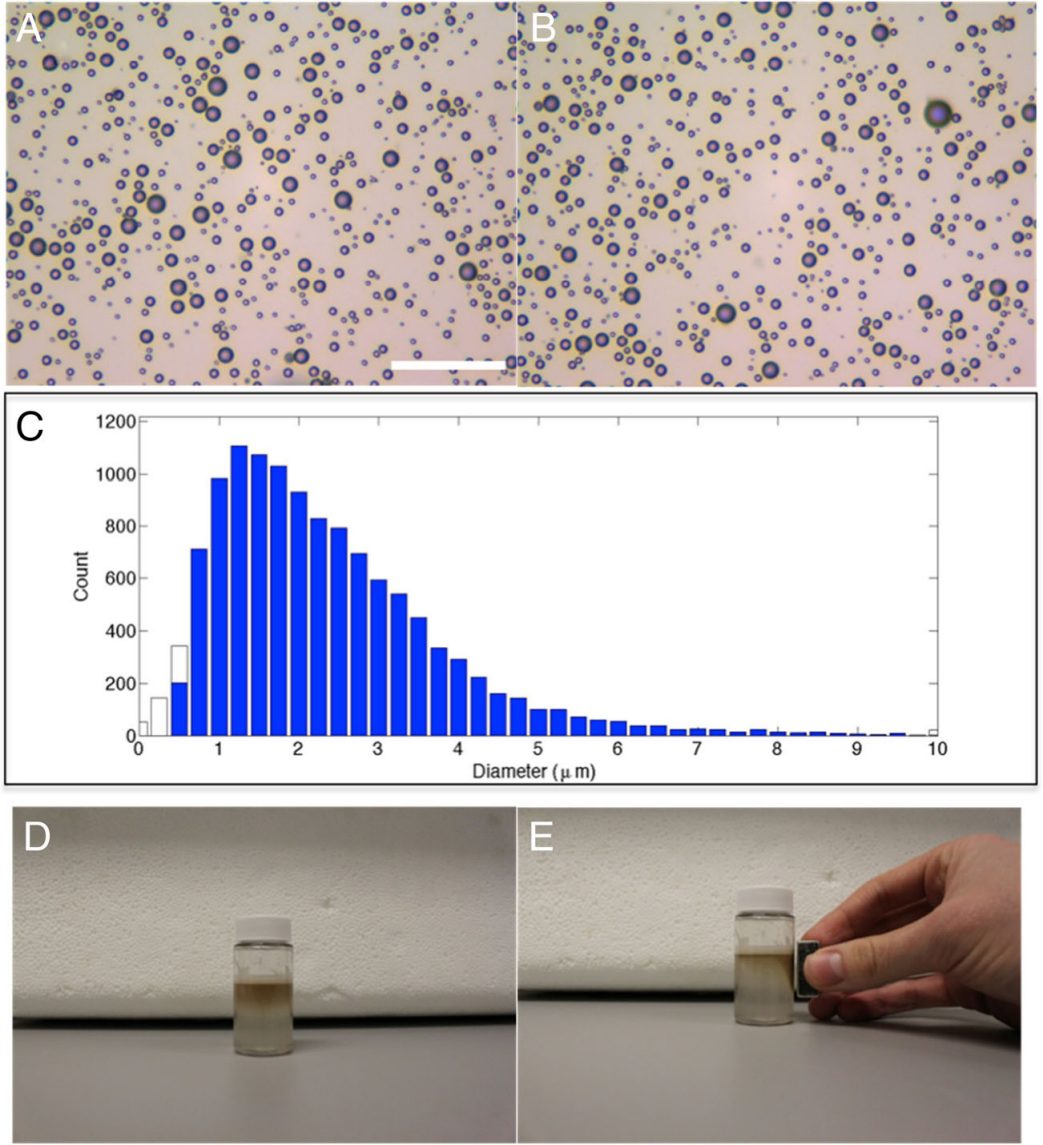
Example of a microscope image of **a** microbubbles (medium chain) and **b** magnetic microbubbles (at ×40 magnification, *scale bar* 50 μm for both images). **c** Size distribution of magnetic microbubbles showing the majority of microbubbles are within the clinically relevant size range <8 μm. A vial of magnetic microbubbles **d** before and **e** after application of a magnetic field is also shown, with noticeable accumulation of microbubbles in proximity to the magnet

On application of a magnetic field, all the magnetic microbubble formulations were observed to move towards the magnet (Fig. 4d, e), indicating successful incorporation of the nanoparticles.

**Fig. 5:**
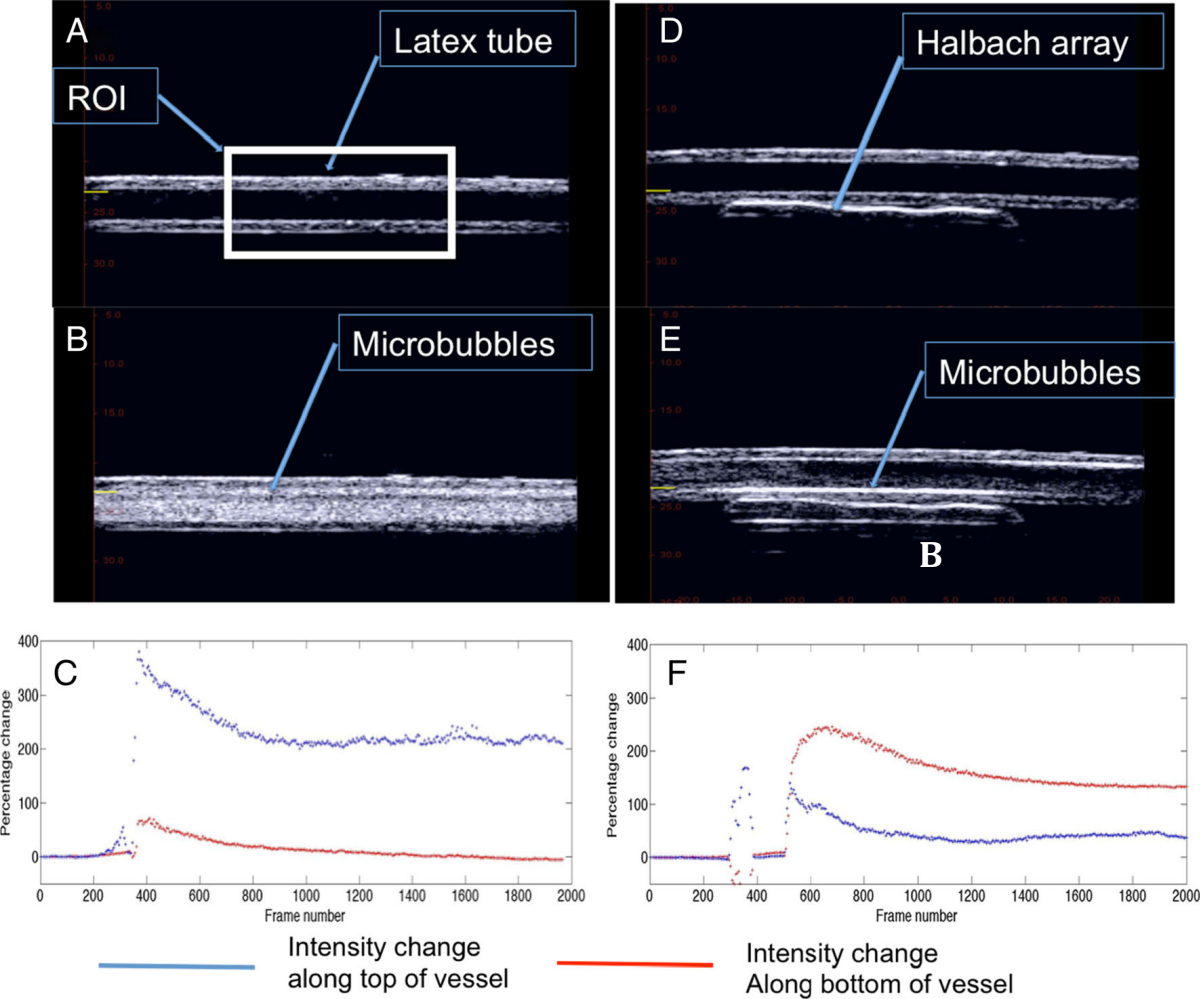
Acoustic images of targeting of magnetic microbubbles. **a** Latex tube with water flowing through. **b** After injection of magnetic microbubbles. **c** Intensity analysis in the region of interest (*ROI*) along the bottom wall (*red*) of the vessel and the top wall (*blue*) indicates the highest signal intensity was detected at the top of the tube over the course of the ultrasound. **d** The same latex tube with a magnetic Halbach array positioned underneath with water flowing through. **e** After injection of magnetic microbubbles, an increase in signal intensity is observed along the bottom of the tube. **f** Intensity analysis within the same ROI along the top (*blue*) and bottom (*red*) walls of the tube indicates that the highest intensity occurred at the bottom of the tube at the wall closest to the magnetic Halbach array

Magnetic microbubbles were also injected into a latex vessel under flow with and without a magnetic field applied to a region of the tube in which the microbubbles were imaged. Magnetic microbubbles flowed throughout the entire vessel when no magnetic field was applied as shown in Fig. 5a, b.

When a magnetic Halbach array was inserted beneath the latex vessel, an increase in signal intensity was observed along the wall of the vessel closest to the Halbach array (Fig. 5d, e). In order to confirm magnetic targeting had occurred, the videos were processed using MATLAB to quantify the increase in intensity. When no magnetic field was applied, the microbubbles moved to the top of the vessel owing to buoyancy. The wall furthest from the Halbach array thus showed a large increase in signal intensity as shown in Fig. 5c. When the Halbach array was inserted, the signal intensity at the wall closest to the magnet increased, indicating that the microbubbles had responded to the magnetic field (Fig. 5f). This test was based on a previous extensive study of magnetic targeting of microbubbles and as such one flow condition was selected to test the targeting ability against flow [53]. This indicates that microbubbles incorporating magnetic nanoparticles in the shell respond to a magnetic field and can target against the flowing blood in the human body.

### Magnetic relaxivity

**Fig. 6:**
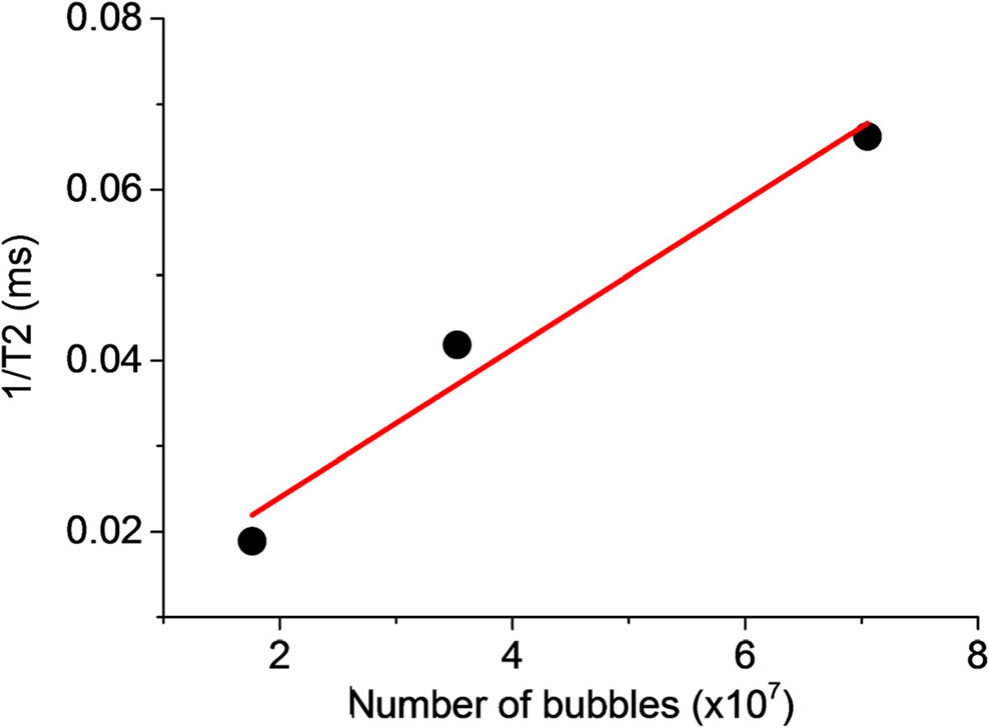
Magnetic relaxivity of magnetic microbubble formulation created with lipid-coated magnetic nanoparticles for three different concentrations of microbubbles

A vibrating sample magnetometer (VSM) was used to confirm that the microbubbles were superparamagnetic (Supplementary Fig. 2). The results in Fig. 6 show that the measured magnetic relaxivity of the microbubble formulation was 0.9 × 10^6^ bubbles/s. This result was performed on a suspension that had been centrifuged (300 RCF, 10 min) to remove unbound magnetic nanoparticles. Based on the relaxivity values for existing contrast agents, the results indicate that the magnetic microbubble formulation would provide MRI contrast enhancement [57].

### Transmission electron microscopy

**Fig. 7:**
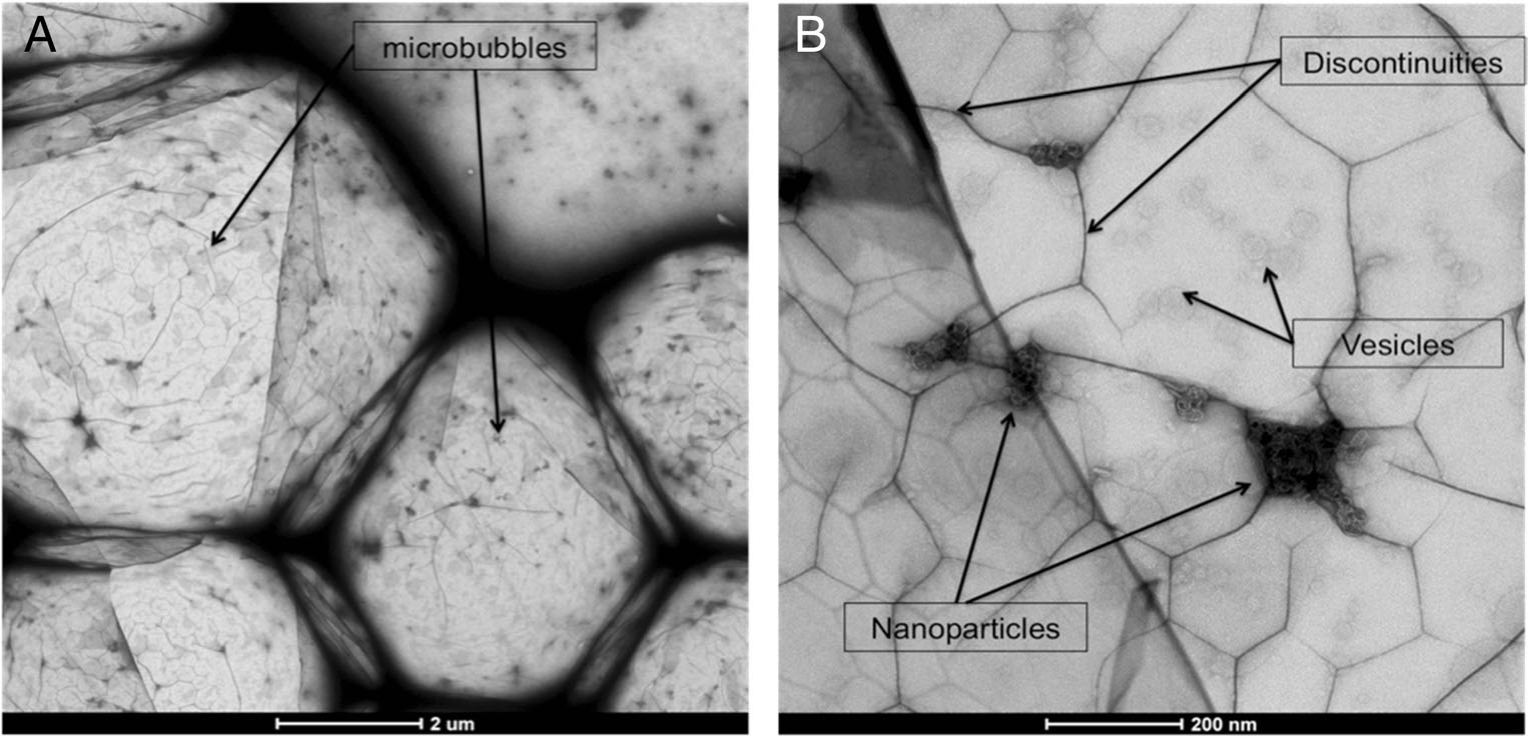
Transmission electron microscopy images of DSPC/PEG-40 stearate (9:1 M ratio) microbubbles created with lipid-coated nanoparticles. **a** The whole structure and size of magnetic microbubbles. **b** Increased magnification of the microbubble shell

Microbubbles were observed under electron microscopy (Fig. 7a), allowing the nanoscale features of the shell to be analysed. Clusters of lipid-coated nanoparticles were seen to be incorporated into the shell (Fig. 7b). This, combined with the magnetic response of the microbubbles, indicates that sufficient quantities of nanoparticles were incorporated into the microbubbles to facilitate targeting and/or MR imaging. The nanoparticles have a nominal hydrodynamic diameter of 50 nm, but the geometric diameter appears to be much smaller, approximately 20 nm. The nanoparticles were clustered at discrete points within the shell. This appeared to cause additional discontinuities in the shell structure as compared to microbubbles without nanoparticles observed by Owen and Stride in previous work [52]. Some nanoscale vesicles that did not contain iron oxide were also observed fused or embedded in the microbubble surface (Fig. 7b). There are relatively few nanoparticles visible in the images, which is surprising given the magnetic response of the microbubbles shown in Figs. 4, 5 and 6 above. However, the surface of the shell can only be observed in two dimensions and so the images may not provide an accurate indication of the number of particles actually embedded within the shell. This information provides direct evidence of magnetic nanoparticles incorporating into the microbubble shell. From the images, it is possible to determine that there are approximately 167 ± 98 NPs/μm^2^ from measuring 10 microbubbles using ImageJ. The total number of nanoparticles on each microbubble can vary from 2000 to 9000 with bubble size as the key determinant. However, it is likely that this is an underestimate as the nanoparticle cluster at sites on the microbubble and the clusters likely have depth in the shell whereas the measurements obtained are only in 2D.

### siRNA attachment

#### Fluorescence microscopy

**Fig. 8:**
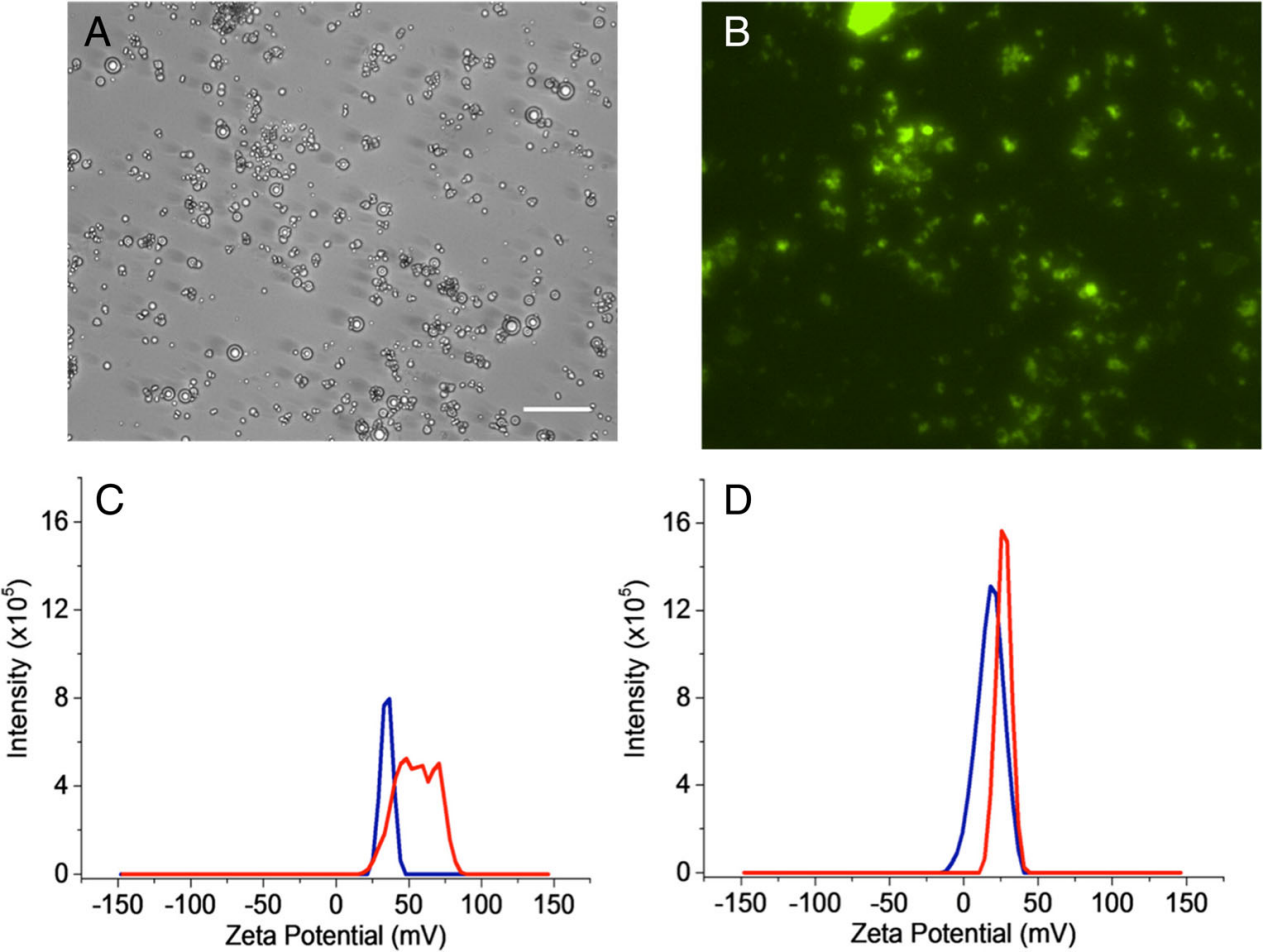
Microscopy images of magnetic microbubbles bound to siRNA after centrifugation in **a** bright field and **b** fluorescence (10-s exposure, gain 1) using a ×20 objective. *Scale bar* is 50 μm. **c** Zeta potential of charged microbubble precursors before (*red*) and after (*blue*) the addition of siRNA. **d** Zeta potential of magnetically charged microbubble precursors before (*red*) and after (*blue*) the addition of siRNA

Charged magnetic microbubbles sonicated with siRNA followed by washing via centrifugation were seen to fluoresce, indicating successful incorporation of the siRNA. The circular shape of the microbubbles could also be discerned in the images as shown in Fig. 8a, b.

#### Zeta potential

As microbubbles are buoyant and will float during the zeta potential measurements, this limits the amount of time over which the measurement can be performed and reduces the accuracy of the measurement. Lipid vesicles (microbubble precursors before sonication at the gas–water interface) are neutrally buoyant and can be measured more accurately for longer periods of time. As microbubbles and vesicles were found to have the same zeta potential, examination of siRNA attachment was performed on vesicles. It was found that magnetic vesicles had a lower zeta potential than non-magnetic vesicles. However, the zeta potential was still positive, allowing negatively charged siRNA to bind to it. Upon the addition of siRNA to the vesicle mixtures, the zeta potential was found to have decreased. This occurred for magnetic and non-magnetic vesicles as shown in Fig. 8c, d. This reduction in zeta potential indicated that siRNA had successfully bound to the vesicle surface and that the magnetic nanoparticles did not impair the attachment process. The fact that the zeta potential remained positive suggests that the loading efficiency may not have been maximised and that the quantity of DSEPC and/or siRNA to the microbubble ratio could be optimised to improve this.

### siRNA delivery

#### Fluorescence microscopy

In order to confirm whether or not siRNA was taken up by the cells, fluorescence microscopy images were obtained (Supplementary Fig. 3E-H). All locations on each OptiCell™ were analysed. A fluorescence intensity increase was observed at all locations where magnetic microbubbles, ultrasound and a magnetic field were applied. However, the first two insonation locations showed the largest increase in fluorescence intensity.

**Fig. 9:**
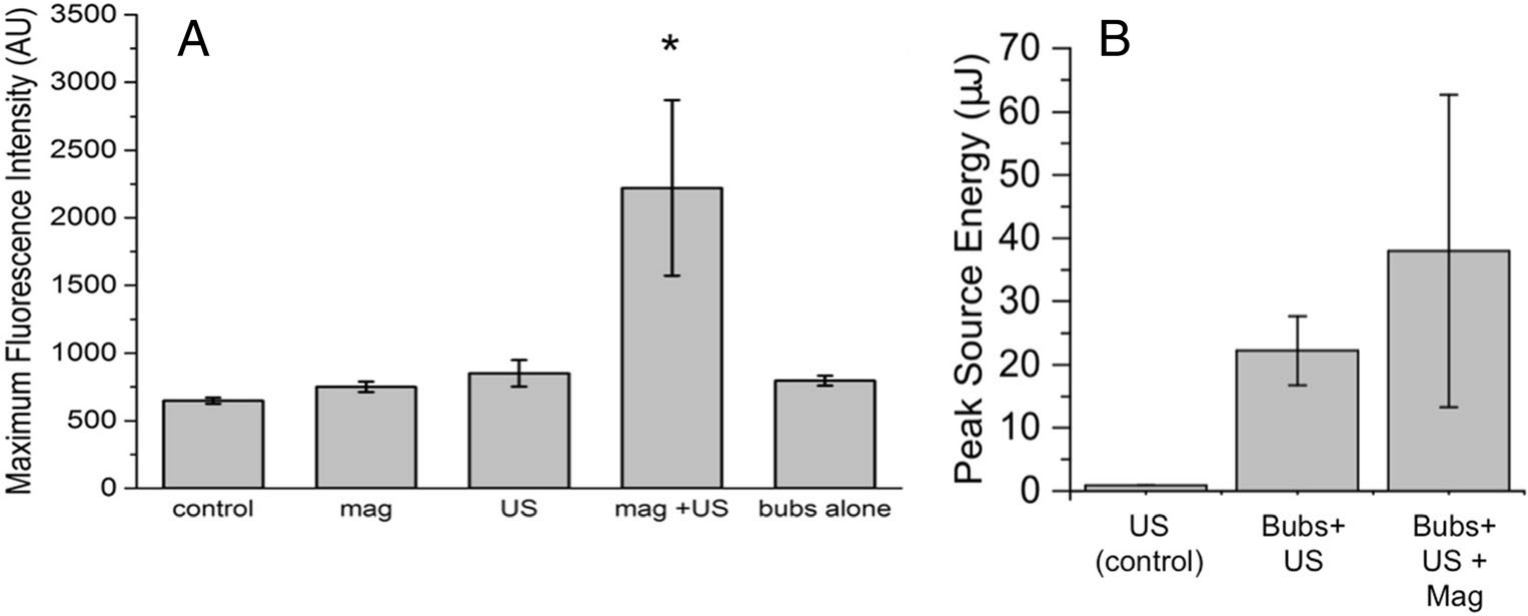
**a** Bar chart of the average maximum fluorescence intensity from all microscope images of cells obtained. Control is cells without exposure to siRNA, and all other results are after exposure to microbubbles with siRNA with the conditions listed underneath. *Error bars* indicate standard deviation (*n* = 6, except for mag where *n* = 3; *p < 0.01, with all other groups). **b** Summary of PAM data. The mean and standard deviation of the peak value in each map over the six treatment locations are shown. *US* ultrasound, *mag* magnetic field, *bubs* magnetic microbubbles

All the images obtained from each OptiCell™ were then analysed for fluorescence intensity by ImageJ in order to obtain the maximum fluorescence for each image (Fig. 9a). Only the combination of a magnetic field and ultrasound showed a statistically significant difference in fluorescence. The analysis of variance (ANOVA) statistical test was performed giving a *p* value of <0.01. Using Tukey’s test, a significant difference (*p* < 0.01) was observed between the group, where a magnet and ultrasound (mag + US) were applied, and all other groups.

#### Passive acoustic mapping

Acoustic maps showing the emissions from each of the ultrasound-treated samples are shown in Supplementary Fig. 4. In each sample, maps showing the distribution of acoustic emissions are displayed with the six treatments per sample arranged corresponding to their position in the OptiCell™ and order of successive treatments shown by the arrows. The focus of the transducer (and region in which cavitation is expected) is located approximately in the centre of each map.

These data are summarised in Fig. 9b. In the sample without magnetic microbubbles, cavitation energy was consistently low over the six exposures (mean 0.87 ± 0.02 μJ) due to the lack of cavitation nuclei. In the sample with magnetic microbubbles and ultrasound (without magnet), the energy of cavitation increased by a factor of 25 (mean 22.2 ± 5.5 μJ). In the sample with magnetic microbubbles, ultrasound and a magnetic field, the mean cavitation energy increased by a further 70% (mean 38.0 ± 24.7 μJ) over the non-magnet case, while the maximum energy of cavitation more than doubled (67.7 vs. 30.9 μJ). Within each map, cavitation (where present) occurred at the expected location close to the transducer focus. Comparing the six maps per sample shows (Supplementary Fig. 4) that in the absence of magnetic targeting, cavitation appears to be relatively evenly distributed over the area of the chamber, while when the magnet was added, cavitation was more localised. Cavitation in the locations treated first was of greater amplitude than that without targeting and showed a pronounced decline on subsequent exposures. These findings are in agreement with the fluorescence intensity data described above.

#### Plate reader results

After the OptiCell™ samples were exposed to the conditions outlined above, they were analysed in the plate reader. The data further support the microscopy images as OptiCell™ 3, to which both ultrasound and a magnetic field were applied, shows the highest fluorescence increase across the plate (Supplementary Fig. 3A-D). The first two spots also show the highest concentration of fluorescent material corresponding to the passive acoustic mapping (PAM) data. However, final spot 6, in the top left-hand corner (Supplementary Fig. 2C), shows an increase in fluorescence which does not correspond to an increase in acoustic activity. This was unexpected and is believed to be due to the first exposure, distributing siRNA over a wide area. However, this requires further investigation. The plate reader results, combined with the fluorescence microscopy and passive acoustic maps, indicate that magnetically targeted microbubbles cavitating under ultrasound exposure delivered fluorescent siRNA to the cells in the target area.

## Concluding remarks

In this paper, a method for incorporating nanoparticles into phospholipid microbubble formulations was presented. It was shown that the method can be applied to a variety of lipid bubble formulations with no additional processing steps required and that the particle-loaded microbubbles can be generated with a size distribution and stability appropriate for intravenous administration. It was further shown that the magnetic microbubbles fabricated in this study could be targeted against flow using a magnetic field, potentially utilised as contrast agents for MRI and used to deliver siRNA to a cancer cell line. This technique could be adapted for other lipid-coated nanoparticles such as gold- or drug-encapsulating particles without requirement for charge or biomimetic techniques allowing easy adaptation of current clinically approved phospholipid microbubbles. It could also provide more information on the process of microbubble formation and the structure of the coating.

## Electronic supplementary material

Supplementary Figure 1 (DOCX 209 kb)

Supplementary Figure 2 (DOCX 38 kb)

Supplementary Figure 3 (DOCX 454 kb)

Supplementary Figure 4 (DOCX 195 kb)

## Footnotes

1 The purpose of these tests was to demonstrate the delivery potential of the microbubbles. As mentioned in the “Introduction” section, the use of charged bubbles may have disadvantages for in vivo use and an alternative coupling strategy for the therapeutic molecule would be desirable. The development of an alternative coupling strategy was, however, outside the scope of the present study.
