# Supplementary material for: A versatile method for the preparation of particle-loaded microbubbles for multimodality imaging and targeted drug delivery
Source: Drug Deliv Transl Res. 2017 Mar 15;8(2):342–56. doi: 10.1007/s13346-017-0366-7 (PMC5830459; doi:10.1007/s13346-017-0366-7)
Supplement: Supplementary file 1 — (DOCX 209 kb) [file 13346_2017_366_MOESM1_ESM.docx]

**Supplementary Figures**


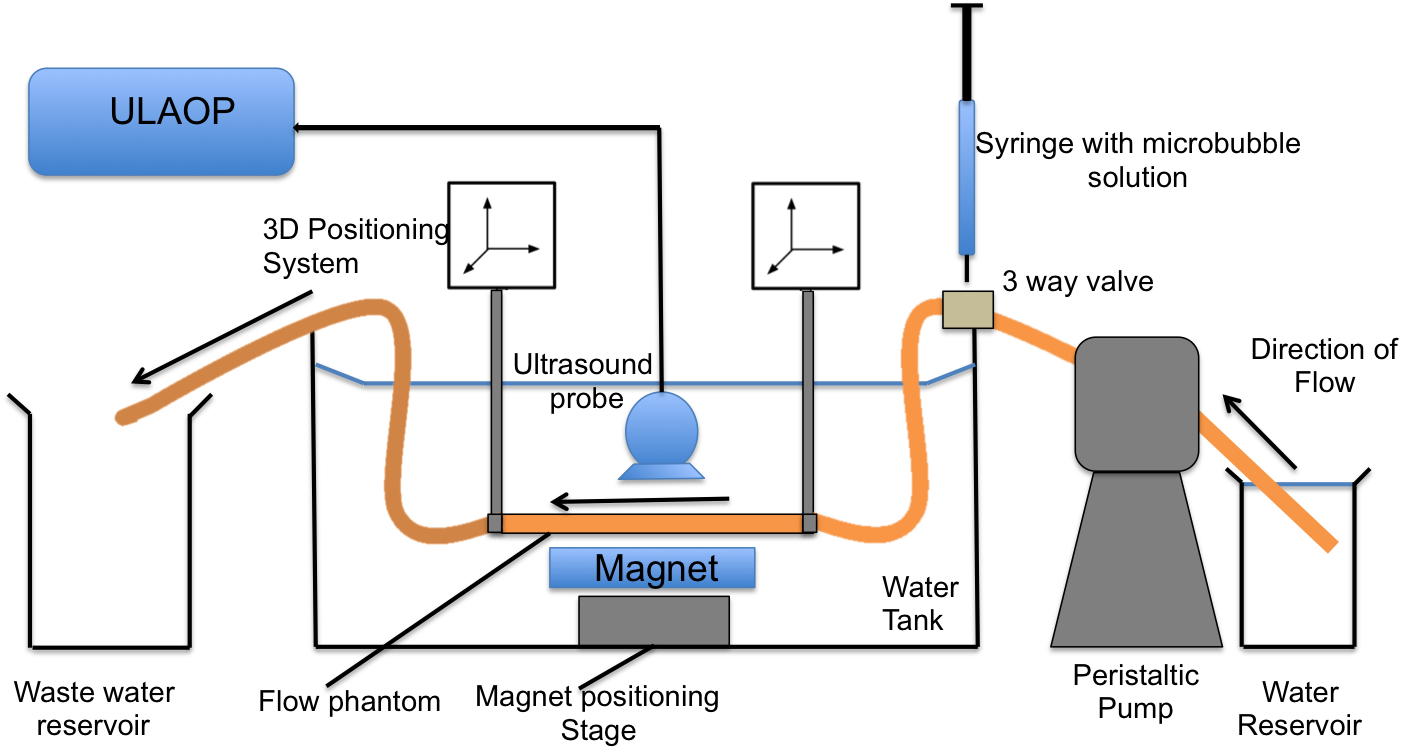


**Supplementary Figure 1; schematic of the flow phantom with the magnetic Halbach array inserted below**
