# Supplementary material for: A versatile method for the preparation of particle-loaded microbubbles for multimodality imaging and targeted drug delivery
Source: Drug Deliv Transl Res. 2017 Mar 15;8(2):342–56. doi: 10.1007/s13346-017-0366-7 (PMC5830459; doi:10.1007/s13346-017-0366-7)
Supplement: Supplementary file 2 — (DOCX 38 kb) [file 13346_2017_366_MOESM2_ESM.docx]

Supplementary Figure 2, M-H Curve for cleaned magnetic microbubbles obtained using a MPMS superconducting Quantum Interference Device (SQUID) magnetometer (Quantum Design, Inc., San Diego, CA) at 90 K between +/-5000 Oe.
