# Supplementary material for: A versatile method for the preparation of particle-loaded microbubbles for multimodality imaging and targeted drug delivery
Source: Drug Deliv Transl Res. 2017 Mar 15;8(2):342–56. doi: 10.1007/s13346-017-0366-7 (PMC5830459; doi:10.1007/s13346-017-0366-7)
Supplement: Supplementary file 3 — (DOCX 454 kb) [file 13346_2017_366_MOESM3_ESM.docx]

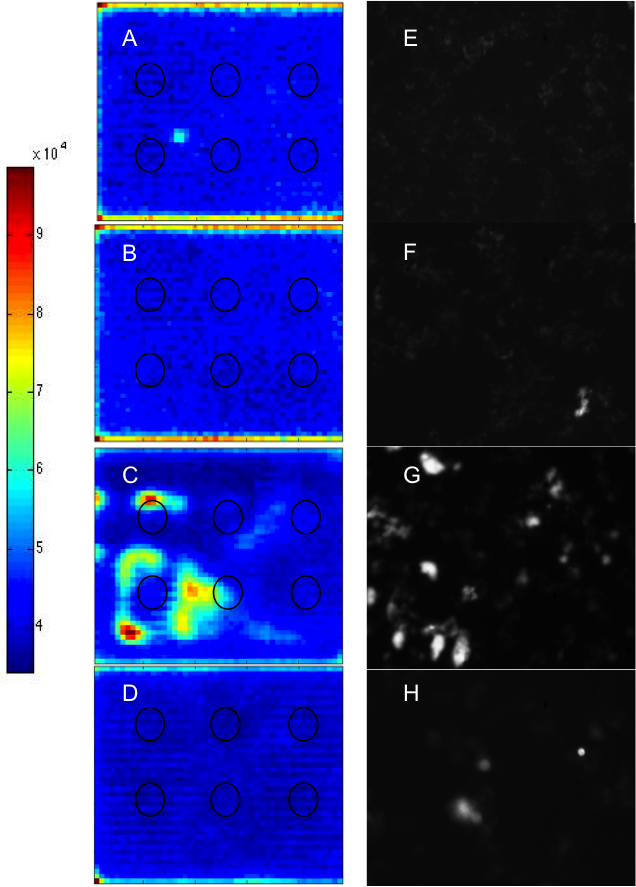


Supplementary Figure 3, Heat maps (A-D) and fluorescence microscopy images (E-H) of fluorescent siRNA having been delivered to SH-SY5Ycells in an Opticell^TM^. Heat maps are of the entire Opticell^TM^, fluorescence microscopy images are a 5 mm x 5 mm of a single insonation location. Opticell^TM^ 1 (A,E) shows cells after exposure to microbubbles alone, Opticell^TM^ 2 (B,F) exposure to microbubbles in the presence of a magnetic field, Opticell^TM^ 3 (C,G) exposure to microbubbles, a magnetic field and ultrasound and Opticell^TM^ 4 (D,H) shows exposure to microbubbles and ultrasound. The colour bar represents fluorescence intensity (arbitrary units). The six black circles represent areas of ultrasound application.
