# Supplementary material for: A versatile method for the preparation of particle-loaded microbubbles for multimodality imaging and targeted drug delivery
Source: Drug Deliv Transl Res. 2017 Mar 15;8(2):342–56. doi: 10.1007/s13346-017-0366-7 (PMC5830459; doi:10.1007/s13346-017-0366-7)
Supplement: Supplementary file 4 — (DOCX 195 kb) [file 13346_2017_366_MOESM4_ESM.docx]

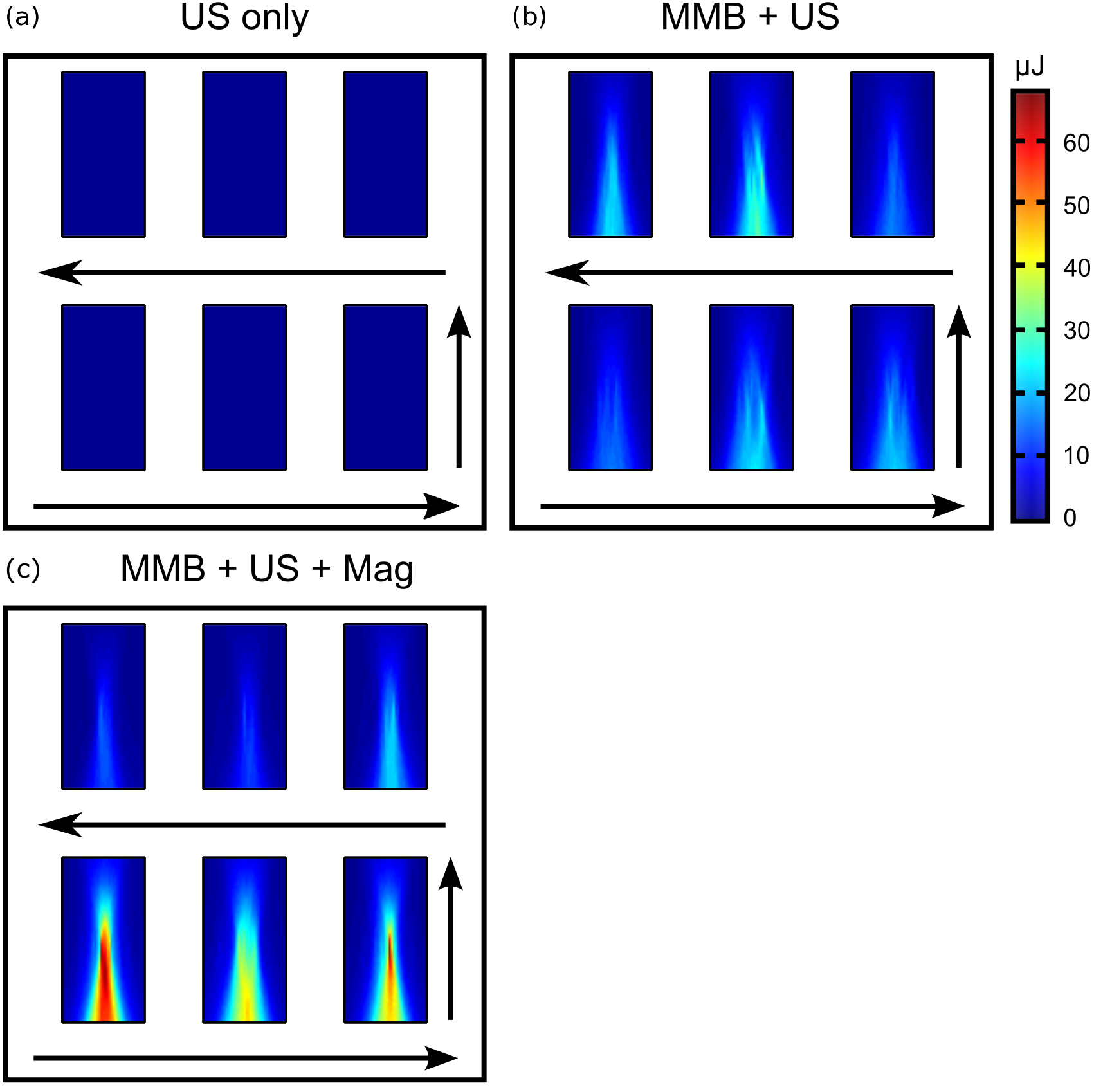


Supplementary Figure 4, (A-C) Passive acoustic mapping (PAM) data for transfection experiments with ultrasound. Each sample was treated six times in the order indicated by the arrows, PAMs show a cross section through the sample. Each map shows a region of interest of ±10 mm transverse and ±20 mm axial with respect to the focus of the transducer and the centre of the magnetic array (corresponding to the maximum force), which is located approximately in the middle of the maps. A) Opticell^TM^ alone as a control, b) Opticell^TM^ 4 with magnetic microbubbles and ultrasound. c) Opticell^TM^ 3 with magnetic microbubbles, ultrasound and a magnetic field. The colour bar represents energy.
